# Thermal influence on life history traits and population parameters of the olive moth, *Prays oleae* (Bernard) (Lepidoptera: Praydidae): implications for temperature-based pest management

**DOI:** 10.3389/finsc.2026.1763467

**Published:** 2026-03-13

**Authors:** Mohamed El Aalaoui, Mohamed Sbaghi

**Affiliations:** Department of Plant Protection, National Institute of Agricultural Research, Rabat, Morocco

**Keywords:** Insect physiology, life-history traits, mortality rate, pest management, temperature thresholds, thermal biology

## Abstract

**Introduction:**

The olive moth, *Prays oleae* (Bernard) (Lepidoptera: Praydidae), is a major pest of olive crops worldwide.

**Methods:**

This study examined the effects of constant temperatures (15–35 °C) on its development, survival, and reproduction, and evaluated nine temperature-dependent models (Linear, Lactin-2, β type, Brière-1 and 2, Polynomial, Shi, SSI, and Taylor).

**Results and discussion:**

Developmental duration decreased with temperature, with egg incubation ranging from 14.1 days at 15 °C to 2.6 ± 0.1 days at 35 °C, pre-adult development ranging from 64.9 days (male) and 68.3 days (female) at 15 °C to 27.1 days and 27.8 days at 35 °C, with males generally developing faster than females except at 28 and 32 °C, and adult life span declining from 76.8–81.2 days at 15 °C to 34.1–35.4 days at 32 °C. Survival followed a bell-shaped pattern, peaking at 25 °C for eggs (79.93%), larvae (90.87%), and pupae (66.0%). Kaplan–Meier analysis indicated faster mortality at higher temperatures (LT_50_ = 35 days at 32 °C vs. 79 days at 15 °C). Pupal weight decreased with temperature, with females consistently heavier than males (15 °C: 7.50 vs. 6.40 mg; 32 °C: 4.88 vs. 4.40 mg). Pupal deformities were marginal (3.1–10.8%), whereas adult deformities increased at temperature extremes (14.9–19.8%). Fecundity peaked at 25 °C (380.7 eggs/female), oviposition was longest at 25 °C (12.6 days), and pre-oviposition decreased from 4.9 days (15 °C) to 1.6 days (32 °C). Model evaluation showed Brière-2 provided the most biologically realistic thermal thresholds (*T_L_*  = 4.3–15 °C, *T_opt_* = 28–34 °C, *T_H_*  = 37.8–42.9 °C).

**Conclusion:**

Overall, *P. oleae* develops and reproduces optimally at 25–28 °C, providing critical data for predicting population dynamics and guiding temperature-based management strategies in olive orchards.

## 1 Introduction

The olive moth, *Prays oleae* (1) (Lepidoptera: Praydidae), is a key pest of olive groves in the Mediterranean basin, the Black Sea region, the Middle East, and the Canary Islands (2, 3). Worldwide, it ranks as the second most destructive olive pest after the olive fruit fly, *Bactrocera (Dacus) oleae* (Gmelin) (Diptera: Tephritidae) (4). Being monophagous, it feeds exclusively on the olive tree (*Olea europaea* L.) (5). The species completes three generations per year, each exploiting a different plant structure: leaves, flowers, and fruits (2). The first generation, phyllophagous, extends from autumn to spring, during which larvae develop inside leaf mines to survive winter conditions (4). In spring, they give rise to the anthophagous generation, the shortest phase, where larvae attack flower buds—one larva may consume up to 20–30 flowers (6). The final generation, carpophagous, occurs in summer, when larvae penetrate the fruit and feed on the seeds (7). This stage is the most harmful, as it causes premature fruit drop and substantial yield reductions (4). Economic losses caused by *P. oleae* can be severe. In Andalusia (southern Spain), outbreaks occur roughly every three years, with production losses reaching 50–60% and fruit drop around 40% (4, 8). More broadly, reported yield reductions attributed to this pest range between 49% and 63% (8, 9).

The management of *P. oleae* relies on integrated strategies combining chemical, biological, and cultural measures (10). Biological control is central, particularly the parasitoid *Opius concolor* Szépl. (Hymenoptera: Braconidae), along with the predator *Chrysoperla carnea* (Stephens) and microbial insecticides such as *Bacillus thuringiensis* Berliner (Bacillales: Bacillaceae) (11–13). Complementary tools—such as toxic traps, the ECO-TRAP system, copper-based products, and improved monitoring and modeling—enhance control while reducing reliance on bait sprays (14–16). In Morocco, integrated management combines *B. thuringiensis*, *C. carnea*, and parasitoids such as *Chelonus eleaphilus* Silv. (Hymenoptera: Braconidae), with cultural practices like plowing and ammonium sulfate trapping (12, 17), while in Greece*, B. thuringiensis*, *C. carnea*, and *O. concolor* are key tools for reducing pesticide use (13, 18). However, environmental variability, especially temperature, and difficulties in sampling large areas quickly limit the precise application of these control measures (19).

Temperature is a critical factor influencing the development, survival, reproduction, and population dynamics of ectothermic insects (20). It can also disrupt synchrony between pests and their hosts, alter interspecific interactions, and shift species’ distribution ranges, with implications for invasions (21–23). Understanding temperature effects on insect development is therefore essential for predicting field population dynamics (24). High temperatures above 30 °C reduce the mobility of *P. oleae* larvae and hinder their ability to penetrate olive fruits, although the exact thermal maximum causing mortality for any life stage is still unknown (19). Low temperatures below 10 °C slow the activity of adult *P. oleae*, and mortality can occur below 7 °C (19). Larval and pupal mortality is also closely linked to the number of days with subzero temperatures (25).

Mathematical models are widely used to study insect biology, particularly to understand how development rates respond to temperature (26–28). Insects typically exhibit a non-linear temperature-dependent development pattern: rates increase above the lower thermal threshold (*T_L_*) up to an optimum temperature (*T_opt_*) and then decline to zero at the upper thermal threshold (*T_H_*) (26). Although development peaks at *T_opt_*, this temperature may not be optimal for survival or population growth (29). Some models estimate additional parameters, such as the intrinsic optimum temperature, which better reflect conditions for development, survival, and reproduction (30). Because thermal thresholds vary among species, selecting an appropriate model to describe temperature-dependent development is crucial (31). Once a suitable model is chosen, insect development can be simulated under field conditions using temperature time series data (32). Timing of control measures is critical because insecticides are often more effective against specific life stages; poor timing can lead to ineffective pest management (29, 33). Accurate phenology models help align control measures with vulnerable pest stages, reducing pesticide use and environmental impact while providing economic benefits (33, 34). This approach has been successfully applied to multiple agricultural and forest pests (29, 33).

Although the effects of temperature on *Prays oleae* development and reproduction have been previously studied (19, 35, 36), these studies relied on artificial diets and narrow temperature ranges, limiting the use of non-linear models (29). Moreover, thermal thresholds were often estimated using linear regression despite the insect’s non-linear response to temperature (36). With *P. oleae* becoming a major olive pest under climate change, its temperature-dependent development remains poorly understood, particularly regarding the selection of appropriate mathematical models. This study therefore aimed to evaluate the effects of temperature on the development, survival, and reproduction of *P. oleae* and to identify suitable models describing its temperature-dependent development rates.

## 2 Materials and methods

### 2.1 Insect collection and rearing

**Figure 1:**
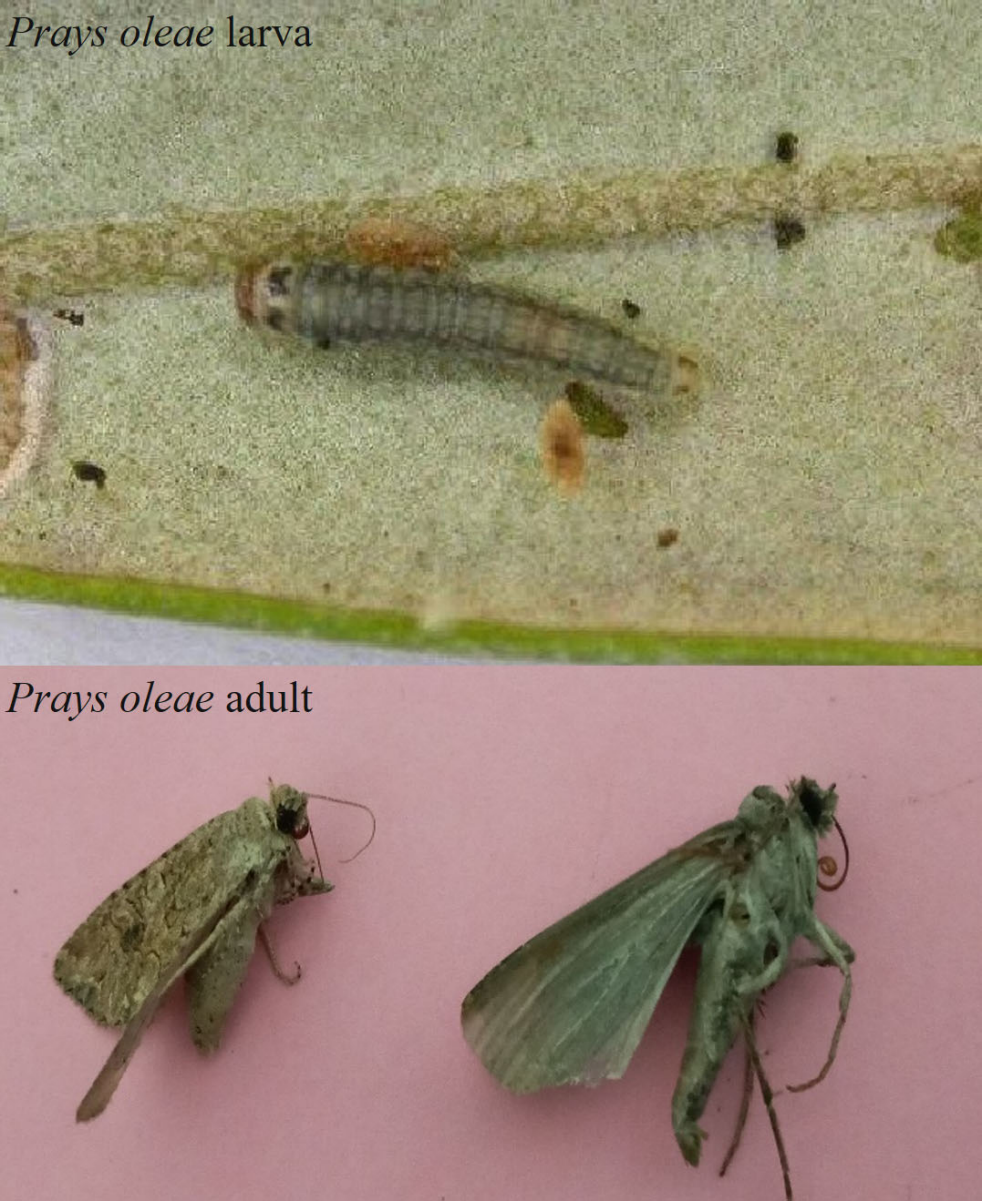
Morphological characteristics of *Prays oleae* showing the larval stage on leaf tissue and the adult moth.

Larvae and pupae of *P. oleae* were collected from olive orchards in Zemamra, Morocco (32°37′48″ N, 8°42′0″ W; 165 m a.s.l.). Collected larvae were transferred to the laboratory and maintained in mesh cages (50 × 50 × 50 cm), where they were supplied daily with fresh, pesticide-free olive leaves. Pupae obtained from the cages, along with a single batch of pupae collected directly from the field, were transferred into new mesh cages with the same characteristics (50 × 50 × 50 cm) until adult emergence. All experimental insects were reared under laboratory conditions for three consecutive generations (G_1_–G_3_) before being used in experiments, and no additional insects were collected from the field. Adults were provided with a cotton pad soaked in 10% sugar solution, which was refreshed every two days. For oviposition, pesticide-free olive shoots and leaves were introduced into the cages and replaced daily to collect freshly laid eggs. These eggs were then used in subsequent experiments. Voucher specimens of *P. oleae* were identified following 1 and deposited in the insectarium of the National Institute of Agricultural Research (INRA), Zemamra (**Figure 1**).

### 2.2 Effect of temperature on the development and survival of *Prays oleae*

Eggs obtained from the stock colony (G_3_) were used to assess the effects of temperature on the development and survival of *P. oleae*. Individuals were kept from egg to adult stage in temperature-controlled chambers at constant temperatures of 15, 20, 25, 28, 32, and 35 ± 1 °C, 60 ± 10% RH, and a 12:12 h light/dark photoperiod. Each temperature was maintained in a separate, independently controlled chamber, and each chamber constituted one independent experimental unit. Temperature treatments were not alternated within the same chamber during the experiment. Each temperature chamber represented an independent experimental unit, and individuals within chambers were treated as independent biological replicates. Egg development was evaluated at all tested temperatures, including 35 °C, to determine embryonic tolerance to high-temperature stress. However, although eggs hatched at 35 °C, larvae failed to survive beyond the early stage. Because complete post-embryonic development could not be obtained at 35 °C, this temperature was excluded from larval, pupal, and total immature development analyses, as well as from stage-specific temperature–development modeling where full life-stage data were required. To assess the effects of temperature on embryonic development, 20 newly laid eggs, aged < 12 h, were separated using a fine-tipped brush and kept in 80-mL plastic vials lined with paper towels. This procedure was repeated 15 times for each temperature evaluated. Each vial constituted one independent replicate, and no vial was reused across temperature treatments to avoid pseudoreplication. The number of hatched larvae was recorded daily to determine the incubation period and egg survival rate. The survival and development of larval and pupal stages were evaluated using 20 newly hatched larvae (< 12 h), individually placed in 150-mL plastic vials for each temperature. This procedure was repeated 15 times for each temperature evaluated. Larvae were reared individually to ensure statistical independence and to avoid pseudoreplication. Each larva represented one independent biological replicate, and individuals were maintained exclusively within their assigned temperature chamber throughout development. Larvae were provided with fresh, pesticide-free olive leaves (cv. *Picholine marocaine*), the preferred host plant. Food was replaced daily, rearing vials were cleaned, and surviving individuals and their developmental stage were recorded. The survival and development time of immature stages, pupal weight, and the presence of deformities in pupae and adults were used as biological parameters to assess the effects of temperature on *P. oleae* development. Pupae were sexed by examining the terminal abdominal segments under a stereomicroscope: females were identified by the presence of a broad, slit-like genital opening on the ventral side of the ninth abdominal segment, while males were recognized by a smaller, rounded anal opening and the presence of claspers or genital papillae (35). Each pupa was weighed individually using an analytical balance (Sartorius, Germany) and inspected for deformities 48 h after pupation. Pupae were considered deformed when showing elongated abdominal segments, abnormal wing development, or atypical head structures (29). Adults with malformed wings or those unable to emerge from their pupal exuviae were also recorded as deformed.

### 2.3 Effect of temperature on reproduction of *Prays oleae*

Newly emerged adults (<24 h old) obtained from the stock colony were used to assess the effects of temperature on reproduction. Adults were reared at permissive temperatures (15–32 °C) but then exposed to 35 °C for reproduction experiments to evaluate adult performance under high-temperature stress. Although 35 °C did not allow successful completion of immature development, it was included in the reproductive experiment to evaluate adult performance and reproductive capacity under acute high-temperature stress conditions. Reproductive experiments were conducted in separate, independently controlled temperature chambers (15, 20, 25, 28, 32, and 35 ± 1 °C), each considered an independent experimental unit. Each pair of male and female was placed in a plastic container (11 × 7 × 3 cm) containing a cotton pad soaked in 10% sugar solution and a pesticide-free olive leaf (cv. *Picholine marocaine*) as an oviposition substrate. If a male died before the female, he was replaced with a healthy male of the same age to maintain continuous mating. Each mating pair constituted one independent biological replicate, and containers were not transferred between temperature treatments. Containers were maintained at constant temperatures of 15, 20, 25, 28, 32, and 35 ± 1 °C, 60 ± 10% RH, and a 12:12 h light/dark photoperiod. Each temperature treatment was replicated 15 times. Oviposition substrates were replaced daily, and the number of eggs laid by each female was recorded until her death. Eggs from each female were collected, placed individually in small containers, and monitored for hatching to determine egg viability (eggs that successfully hatched into larvae). Reproductive parameters, including pre-oviposition period, oviposition period, post-oviposition period, total fecundity (eggs per female), and egg hatch rate, were used to evaluate the effects of temperature.

### 2.4 Selection and evaluation of mathematical models

The development durations of *P. oleae* at the different tested temperatures were used to determine the thermal thresholds and to model the temperature–development relationship. One linear and eight non-linear models, previously reported in the literature, were applied to the observed development rates (1/development time) for each immature stage (egg, larva, pupa) and for the total life cycle duration (**Table 1**). Only temperature levels with successful development were included for each stage-specific model. The models were fitted in the R environment using R software version 4.3.2, following the method described by Ikemoto et al. (41).

**Table 1:** Mathematical models used to describe the temperature-dependent development rate of *Prays oleae*.

Model	Function^a^	Reference
Linear	D(T)=a+bT	37
Lactin-2	$D(T)=e^{\left(\rho T\right)}-e^{\left(\rho T_{L}-\frac{T_{H}-T\;}{\Delta }\right)}+\lambda$	27
β type	$1/D=\rho \times (\alpha \times \frac{T}{10})\times {\left(\frac{T}{10}\right)}^{\beta }$	38
Brière-1	$D(T)=aT(T-T_{L}){\left(T_{H}-T\right)}^{1/2}$	38
Brière-2	$D(T)=aT(T-T_{L}){\left(T_{H}-T\right)}^{1/m}$	38
Polynomial	$D(T)=a{\left(T\right)}^{3}+b{\left(T\right)}^{2}+C(T)+d$	39
Shi	$D(T)=m(T-T_{L})[1-e^{k(T-T_{H})}]$	40
SSI	$D(T)=\frac{\rho_{\emptyset }\;\times \frac{T}{T_{\emptyset }}\;\times exp[\frac{\Delta H_{A\;}}{R}(\frac{1}{T_{\emptyset }}-\frac{1}{T})]}{1+exp[\frac{\Delta H_{L\;}}{R}(\frac{1}{T_{L}}-\frac{1}{T})]+exp[\frac{\Delta H_{H\;}}{R}(\frac{1}{T_{H}}-\frac{1}{T})]}$	41
Taylor	$D(T)=Rm\{-0.5{\left[\frac{\left(T-T_{opt}\right)}{T_{L}}\right]}^{2}\}$	42

a *T* is the ambient temperature (°C), and *D(T)* is the development rate at that temperature, expressed as the reciprocal of development time (days⁻¹). *T_L_* and *T_H_* are the lower and upper temperature thresholds, respectively, below or above which development ceases, while *T_opt_* is the temperature at which development is maximal. In the SSI model, *T_ϕ_* is the intrinsic optimum temperature for enzyme activity (K), and ρ_ϕ_ is the corresponding maximum development rate assuming no enzyme inactivation. Other parameters (*a, b, c, d, m, α, β, λ, ρ, Ψ, Δ, Rm*) are empirical coefficients fitted to experimental data. Thermodynamic constants in the SSI model include *ΔH_A_* (enthalpy of activation), *ΔH_L_* and *ΔH_H_* (enthalpy changes associated with low- and high-temperature enzyme inactivation), and R, the universal gas constant (8.314 J mol⁻¹ K⁻¹). Models such as Lactin, Logan, and Brière describe non-linear responses near thermal limits, while linear and polynomial models approximate development rate across a limited temperature range.

The linear model was used to estimate the lower developmental threshold (T_L_) and the thermal constant (K), representing the total heat units necessary for *P. oleae* to complete development from one stage to the next. *T_L_* was calculated as the x-intercept (*T_L_* = −a/b), and K as the reciprocal of the slope (*K* = 1/b). Data obtained at 35 °C were excluded from the linear model analysis because eggs failed to develop at this temperature, exceeding the linear range of the temperature–development relationship. For nonlinear models, data at 35 °C were included only for eggs, but excluded for larval, pupal, and total development stages because eggs did not continue development at this temperature.

The non-linear models varied in complexity according to the number of parameters to be fitted. Simple models such as β type and Brière-1 require three parameters, whereas more complex models, including SSI, have seven parameters (**Table 1**). Models were evaluated with consideration of parameter identifiability relative to the number of temperature levels available for each developmental stage. Models such as Brière-1, Brière-2, Lactin-2, Shi, and SSI allow direct estimation of *T_L_*, optimum temperature (*T_opt_*), and upper thermal threshold (*T_H_*), while others estimate only *T_opt_*and *T_H_* (β type), or *T_L_* alone (Linear).

Model performance was evaluated based on goodness-of-fit and the capacity to produce biologically realistic thermal thresholds. Goodness-of-fit was measured using the **s**tandard error of the regression (S), and the overall quality of each model was further assessed with the corrected Akaike Information Criterion (AICc):


$$\text{AICc}=n\text{ln}(\frac{RSS}{n})+2K+(\frac{2K^{2}+2K}{n-k-1})$$

Where *n* corresponds to the number of temperature treatments (mean development rates) included in the model for a given developmental stage, not the number of individual insects or replicates (for eggs, n= 6 (15, 20, 25, 28, 32, and 35 °C), while for larvae, pupae, and the total immature stage, n= 5 (15, 20, 25, 28, and 32 °C) because development did not continue at 35 °C), RSS is the residual sum of squares, and *k* is the number of model parameters. Models were fitted using the mean development rate at each temperature; thus, n represents the number of temperature treatments rather than individual or replicate-level observations. Lower AICc values indicate better model performance. Additionally, ΔAICc was calculated as the difference between each model’s AICc and the lowest AICc for the same developmental stage. Values of ΔAICc ≤ 2.0 indicate comparable performance.

The thermal thresholds considered in this study were the lower (*T_L_*) and upper (*T_H_*) developmental thresholds and the optimum temperature (*T_opt_*). The estimated thresholds were validated against observed data and the known geographical and ecological range of *P. oleae*. Thresholds outside the biologically plausible ranges (6–15 °C for *T_L_*, 25–32 °C for *T_opt_*, and 30–38 °C for *T_H_*) were considered unrealistic. Preference was given to models that provided thermal estimates consistent with *P. oleae* biology.

### 2.5 Statistical analysis

The development time of *P. oleae* (egg, larval, pupal, total immature duration, adult longevity, and total life span) at different temperatures was compared using the Kruskal–Wallis test at a 5% significance level. This non-parametric approach was chosen to avoid assumptions of normality and homoscedasticity. When significant differences were detected, Dunn’s *post hoc* test (P < 0.05) was applied for pairwise comparisons, with P-values adjusted using the Bonferroni correction to account for multiple comparisons. The durations of males and females for each developmental stage were also compared using the Kruskal–Wallis test at the same significance level. The incidence of deformations in pupae and adults (e.g., malformed wings, abnormal pupal shape, or failure to emerge) was compared across temperatures using contingency tables and the chi-square test at a significance level of 5%. Pupal weights were first tested for normality (Shapiro–Wilk test) and homogeneity of variances (Levene’s test). After confirming assumptions, one-way ANOVA was performed to test the effect of temperature on females, males, and the combined mean. *Post hoc* comparisons among temperatures were conducted using Tukey’s LSD test. In addition, pupal weights of males and females within each temperature were compared using a two-sample t-test, with P-values were adjusted to account for repeated t-tests across temperatures. The survival of immature stages (egg, larva, and pupa), as well as the pre-oviposition, oviposition, and post-oviposition periods and total fecundity (eggs per female), were analyzed using generalized linear models (GLMs). For survival, a binomial distribution with a logistic link function was applied and reported R² values correspond to pseudo-R² (McFadden’s R²), reflecting the proportion of deviance explained by the model relative to a null model. F-tests were applied within the Gaussian GLM framework for continuous traits (pre-oviposition, oviposition, post-oviposition, total fecundity, and egg hatch rate) to test temperature effects. Non-Gaussian response variables were analyzed using the appropriate link functions, and R² or F-tests were not interpreted for these models. When significant differences among temperatures were detected, pairwise comparisons were conducted using Tukey’s test at a 5% significance level. Survival curves at each temperature were estimated using the Kaplan–Meier method and compared with the log-rank test. All statistical analyses were carried using R software version 4.3.2 with the MASS package for GLMs and the survival package for Kaplan–Meier analyses. A significance threshold of α = 0.05 was applied for all tests.

## 3 Results

### 3.1 Effects of temperature on the development and survival of *Prays oleae*

Developmental times of the egg (χ² = 53.5; df = 5; P < 0.01), larval (male χ² = 46.4; df = 4; P < 0.01, female χ² = 45.8; df = 4; P < 0.01), and pupal stages (male χ² = 42.4; df = 4; P < 0.01, female χ² = 45; df = 4; P < 0.01), as well as the pre-adult (male χ² = 44.5; df = 4; P < 0.01, female χ² = 46.2; df = 4; P < 0.01) and adult stages (male χ² = 42.5; df = 4; P < 0.01, female χ² = 41; df = 4; P < 0.01) and overall life span (male χ² = 44.5; df = 4; P < 0.01, female χ² = 46; df = 4; P < 0.01), were significantly affected by temperature (**Table 2**). In general, development duration decreased with increasing temperature between 15 and 32 °C. Egg incubation ranged from 14.1 days at 15 °C to 2.6 ± 0.1 days at 35 °C. Although eggs hatched at 35 °C, larvae did not survive beyond the early stage, so this temperature was excluded from post-embryonic development analyses. Larval development was longer in females than males across all temperatures, ranging from 36.3 days (male) and 39.6 days (female) at 15 °C to 14.2 days (male) and 14.4 days (female) at 32 °C. Pupal duration ranged from 14.9 days (male) and 15.3 days (female) at 15 °C to 6.6–7.1 days at 32 °C, with males generally developing faster than females, except at 20 °C and 25 °C, where no significant differences were recorded. Total pre-adult development was shorter at higher temperatures, with males completing development slightly faster than females (64.9 vs. 68.3 days at 15 °C; 27.1 vs. 27.8 days at 32 °C), except at 28 °C and 32 °C. Adult longevity and life span also decreased with increasing temperature, with males living slightly shorter than females at each temperature (adult longevity: 11.3, 10.2, 8.0, 7.3, 6.8 days for males and 13.0, 10.9, 9.1, 8.0, 7.4 days for females at 15, 20, 25, 28, and 32 °C, respectively; total life span: 76.8, 57.8, 41.2, 36.8, 34.1 days for males and 81.2, 60.9, 44.1, 38.4, 35.4 days for females at 15, 20, 25, 28, and 32 °C, respectively). Overall, these results indicate that temperature strongly influences the developmental duration of *P. oleae*, with females generally exhibiting longer pre-adult and total developmental durations than males.

**Table 2:** Developmental duration (mean ± SE) of *Prays oleae* female and male under different constant temperatures (15, 20, 25, 28, 32, and 35 °C ( ± 1 °C)).

Developmental duration (Days)		Temperature (°C)					
		15	20	25	28	32	35
Egg incubation		14.1 ± 0.1A (n=200)	10.2 ± 0.1AB (n=199)	7.4 ± 0.1BC (n=200)	6.7 ± 0.1CD (n=200)	6.4 ± 0.1CD (n=200)	2.6 ± 0.1D (n=200)
Larva	Male	36.3 ± 0.31Ab (n=57)	26.7 ± 0.1ABb (n=67)	18.0 ± 0.1BCb (n=130)	16.0 ± 0.1CDb (n=65)	14.2 ± 0.1Da (n=45)	–
	Female	39.6 ± 0.1Aa (n=60)	28.1 ± 0.1 (n=70)ABa	19.0 ± 0.2 (n=143)BCa	16.5 ± 0.1 (n=69)CDa	14.4 ± 0.1 (n=49)Da	–
Pupa	Male	14.9 ± 0.2Aa (n=20)	11.0 ± 0.1ABb (n=28)	7.8 ± 0.1BCb (n=65)	7.0 ± 0.1Ca (n=28)	6.6 ± 0.1Ca (n=30)	–
	Female	15.3 ± 0.2Aa (n=222)	12.0 ± 0.1ABa (n=36)	8.2 ± 0.1BCa (n=65)	7.2 ± 0.1Ca (n=31)	7.1 ± 0.1Ca (n=29)	–
Pre-adult	Male	64.9 ± 0.6Ab (n=19)	47.7 ± 0.2ABb (n=26)	33.1 ± 0.1BCb (n=63)	29.5 ± 0.3Ca (n=26)	27.1 ± 0.3Ca (n=30)	–
	Female	68.3 ± 0.5Aa (n=21)	50.0 ± 0.2ABa (n=34)	35.1 ± 0.3BCa (n=64)	30.4 ± 0.2CDa (n=29)	27.8 ± 0.2Da (n=29)	–
Adult	Male	11.3 ± 0.2Ab (n=18)	10.2 ± 0.1ABa (n=26)	8.0 ± 0.1BCb (n=52)	7.3 ± 0.2Cb (n=25)	6.8 ± 0.1Cb (n=23)	–
	Female	13.0 ± 0.2Aa (n=20)	10.9 ± 0.1ABa (n=31)	9.1 ± 0.1BCa (n=55)	8.0 ± 0.1Ca (n=27)	7.4 ± 0.2Ca (n=25)	–
Life span	Male	76.8 ± 0.5Ab (n=18)	57.8 ± 0.2ABb (n=26)	41.2 ± 0.2BCb (n=52)	36.8 ± 0.4Cb (n=25)	34.1 ± 0.4CCa (n=23)	–
	Female	81.2 ± 0.5Aa (n=20)	60.9 ± 0.3ABa (n=31)	44.1 ± 0.4BCa (n=54)	38.4 ± 0.2CDa (n=27)	35.4 ± 0.3Da (n=25)	–

a Means followed by the same uppercase letters within rows are not significantly different according to Dunn’s test at a 5% significance level.

b Means followed by the same lowercase letters within columns, comparing the duration of males and females for each developmental stage, are not significantly different according to the Kruskal–Wallis test at a 5% significance level. n = number of surviving individuals per stage and temperature.

**Figure 2:**
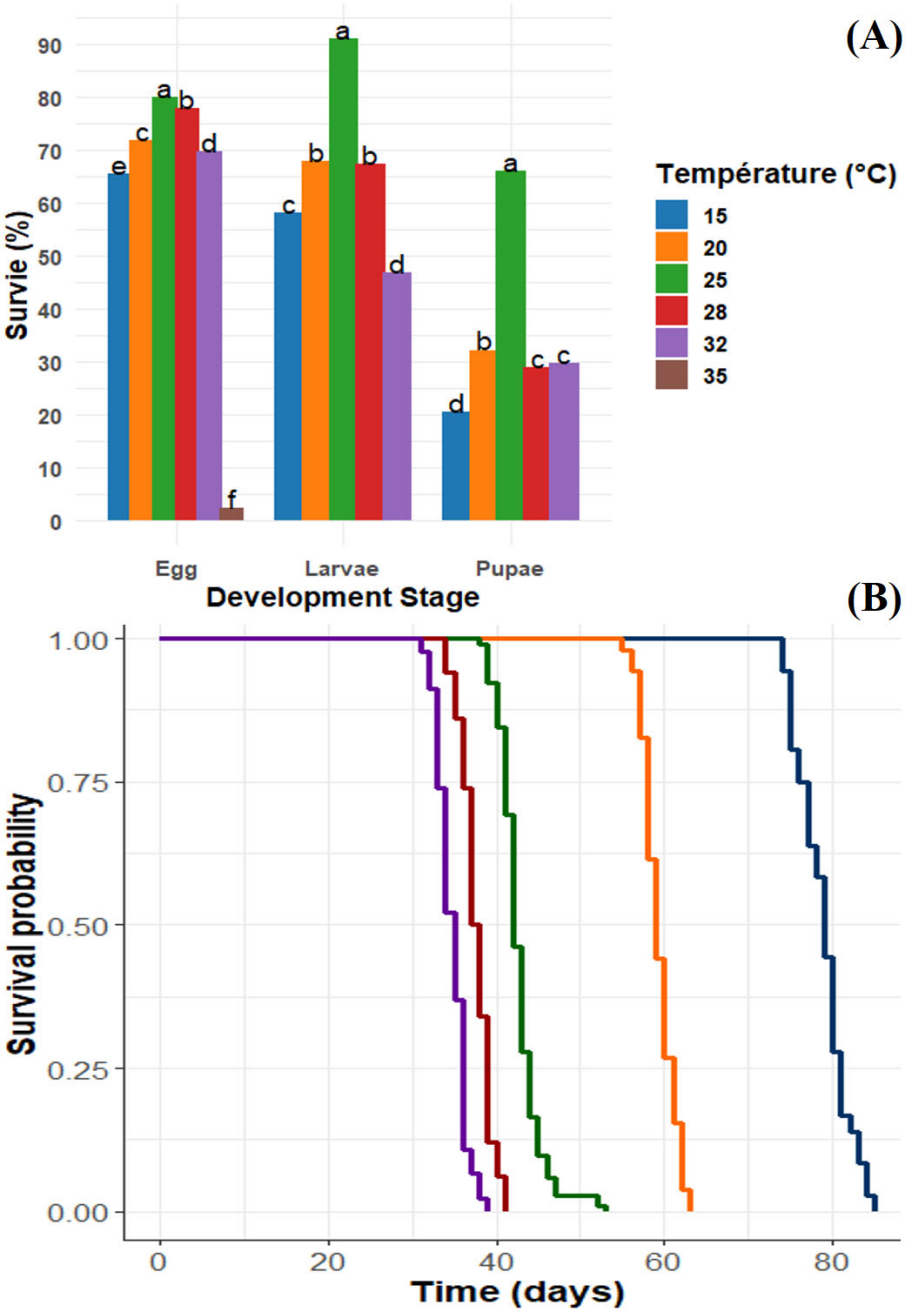
Survival of *Prays oleae* reared at different constant temperature regimes. **(A)** Survival (%) followed by the same lowercase letters in each development stage are not statistically different according to the generalized linear models and Tukey's test at a significance level of 5%.**(B)** Survival curves were generated with non-parametric Kaplan–Meier analysis.

Except for eggs at 35 °C, which hatched but did not continue development, *P. oleae* completed development from egg to adult at all other temperatures tested (**Figure 2A**). Temperature significantly affected survival at all stages. Egg survival was strongly influenced by temperature (F_4_,_84_ = 3535.16, P < 0.001, R² = 0.995), with the highest mean survival observed at 25 °C (79.93%) and the lowest at 15 °C (65.53%) and 32 °C (69.73%). Larval survival also varied significantly across temperatures (F_4_,_70_ = 2141.77, P < 0.001, R² = 0.992), reaching a maximum at 25 °C (90.87%) and a minimum at 32 °C (46.80%). Pupal survival was significantly affected by temperature as well (F_4_,_70_ = 2634.60, P < 0.001, R² = 0.993), with the highest survival at 25 °C (66.00%) and lowest at 15 °C (20.67%). Kaplan–Meier survival analysis confirmed these patterns, showing that survival decreased at extreme temperatures (**Figure 2B**). The median lethal time (LT_50_) and mean survival time were highest at 15 °C (LT_50_ = 79 days; mean survival = 73.6 days) and lowest at 32 °C (LT_50_ = 35 days; mean survival = 33.1 days), indicating faster mortality at higher temperatures. Overall, survival followed a bell-shaped trend, with optimal survival at moderate temperatures (20–25 °C) and increased mortality at temperature extremes.

### 3.2 Effects of temperature on pupal weight and deformities of *Prays oleae*

Pupal weight of *P. oleae* was significantly affected by temperature and sex (**Table 3**). The results showed a significant effect of temperature for females (F_4_,_186_; = 38.73, P < 0.001) and males (F_4_,_208_ = 62.40, P < 0.001), as well as for the combined mean pupal weight (F_4_,_186_; = 83.82, P < 0.001). In all cases, females were significantly heavier than males at each temperature (15 °C (7.50 mg vs 6.40 mg; t = 6.53, df = 57.5, P = 1.87×10⁻^8^), 20 °C (6.94 vs 6.21 mg; t = 4.03, df = 53.2, P = 1.81×10⁻^4^;), 25 °C (6.69 vs 6.08 mg; t = 6.03, df = 91.3, P = 3.51×10⁻^8^), 28 °C (6.39 vs 5.85 mg; t = 2.87, df = 59.6, P = 0.0057), and 32 °C (4.88 vs 4.40 mg; t = 2.12, df = 45.8, P = 0.039). Pupal weight decreased progressively with increasing temperature, with the heaviest pupae recorded at 15 °C and the lightest at 32 °C. The percentage of deformed pupae was marginally affected by temperature (χ² = 9.07; df = 4; P = 0.059), ranging from 3.1% at 25 °C to 10.8% at 15 °C, with intermediate values of 7.7% at 20 °C, 3.8% at 28 °C, and 9.2% at 32 °C. In contrast, adult deformities were strongly influenced by temperature (χ² = 35.44; df = 4; P = 3.77×10⁻^7^), with the highest percentages observed at 15 °C (14.9%), 28 °C (12.4%), and 32 °C (19.8%), and considerably lower values at 20 °C and 25 °C (1.7%). Overall, these results indicate that increasing temperature reduces pupal weight in both sexes while females remain consistently heavier than males at all temperatures (**Table 3**). Although pupal deformities were largely unaffected by temperature, adult deformities increased at both low (15 °C) and high (28–32 °C) temperatures, highlighting the sensitivity of adult morphology to thermal stress.

**Table 3:** Pupal weight (mean ± SE) and percentage of deformed pupae and adults of *Prays oleae* reared at different constant temperatures (15, 20, 25, 28, 32, and 35 °C ( ± 1 °C)).

Temperature (°C)	Pupal weight (mg)			Deformation(%)^a^	
	Female	Male	Mean	Pupae^b^	Adults
15	7.5 ± 0.1Aa (28)	6.4 ± 0.1Ab (31)	7.0 ± 0.1A (28)	10.8 (130) A	14.9 (121) A
20	7.0 ± 0.2Ba (32)	6.2 ± 0.1Bb (39)	6.6 ± 0.1B (32)	7.7 (130) A	1.7 (121) B
25	6.7 ± 0.1BCa (74)	6.1 ± 0.1BCb (78)	6.4 ± 0.1BC (74)	3.1 (130) B	1.7 (121) B
28	6.4 ± 0.1Ca (32)	5.8 ± 0.1Cb (36)	6.1 ± 0.1C (32)	3.8 (130) A	12.4 (121) A
32	4.9 ± 0.2Da (25)	4.4 ± 0.1Db (29)	4.6 ± 0.1D (25)	9.2 (130) A	19.8 (121) A

Values within parentheses are sample sizes.

Values followed by the same uppercase letters within columns for both pupal weight and deformation (%) are not statistically different according to Tukey’s LSD test for pupal weight and chi-square test for deformation across temperatures at a 5% significance level.

Values followed by the same lowercase letters within rows are not statistically different according to two-sample t-test for male vs female pupal weight within each temperature.

### 3.3 Effect of temperature on the reproductive parameters of *Prays oleae*

The reproductive performance of *P. oleae* females was strongly influenced by temperature (**Table 4**). Pre-oviposition duration decreased significantly as temperature increased, ranging from 4.9 ± 0.2 days at 15 °C to 1.6 ± 0.2 days at 32 °C (F_4_,_70_ = 42.92, P < 0.001, R² = 0.710), indicating faster onset of egg-laying at higher temperatures. Oviposition period varied significantly with temperature (F_4_,_70_ = 42.01, P < 0.001, R² = 0.706), being longest at 25 °C (12.6 days) and shortest at 15 °C (8.5 days). Post-oviposition duration showed a minor but significant effect of temperature (F_4_,_70_ = 2.53, P = 0.048, R² = 0.126), ranging from 0.9 ± 0.1 days at 25–28 °C to 1.3 days at 15 °C. Fecundity was strongly influenced by temperature (F_4_,_70_ = 2063.82, P < 0.001, R² = 0.992), reaching a maximum at 25 °C (380.7 eggs/female) and decreasing at lower (209.7 eggs/female at 15 °C) and higher temperatures (299.9 eggs/female at 32 °C). Adults were also exposed to 35 °C to evaluate reproductive performance under high-temperature stress; however, data at 35 °C are not included in **Table 4** because eggs laid at this temperature did not continue development, and therefore stage-specific comparisons could not be made. Overall, reproductive activity followed a bell-shaped trend with optimal performance at moderate temperatures (25–28 °C) and reduced reproduction at temperature extremes.

**Table 4:** Reproductive parameters of *Prays oleae* females at different constant temperatures (mean ± SE).

Temperature (°C)	Pre-oviposition (day)	Oviposition (day)	Post-oviposition (day)	Fecundity (eggs/female)
15 (20)	4.9 ± 0.2 A	8.5 ± 0.2 D	1.3 ± 0.2 A	209.7 ± 0.9 E
20 (31)	3.6 ± 0.2 B	10.3 ± 0.2 C	1.2 ± 0.1 A	295.0 ± 1.6 D
25 (54)	2.5 ± 0.1 C	12.6 ± 0.3 A	0.9 ± 0.1 A	380.7 ± 2.0 A
28 (27)	2.2 ± 0.2 CD	11.8 ± 0.3 B	0.9 ± 0.1 A	365.2 ± 1.6 B
32 (25)	1.6 ± 0.2 D	9.5 ± 0.2 CD	1.1 ± 0.1 A	299.9 ± 1.1 C

Values within parentheses are sample sizes.

Different letters within columns indicate significant differences (GLM, Gaussian link, Tukey’s test, P < 0.05).

### 3.4 Selection and performance of mathematical models

**Figure 3:**
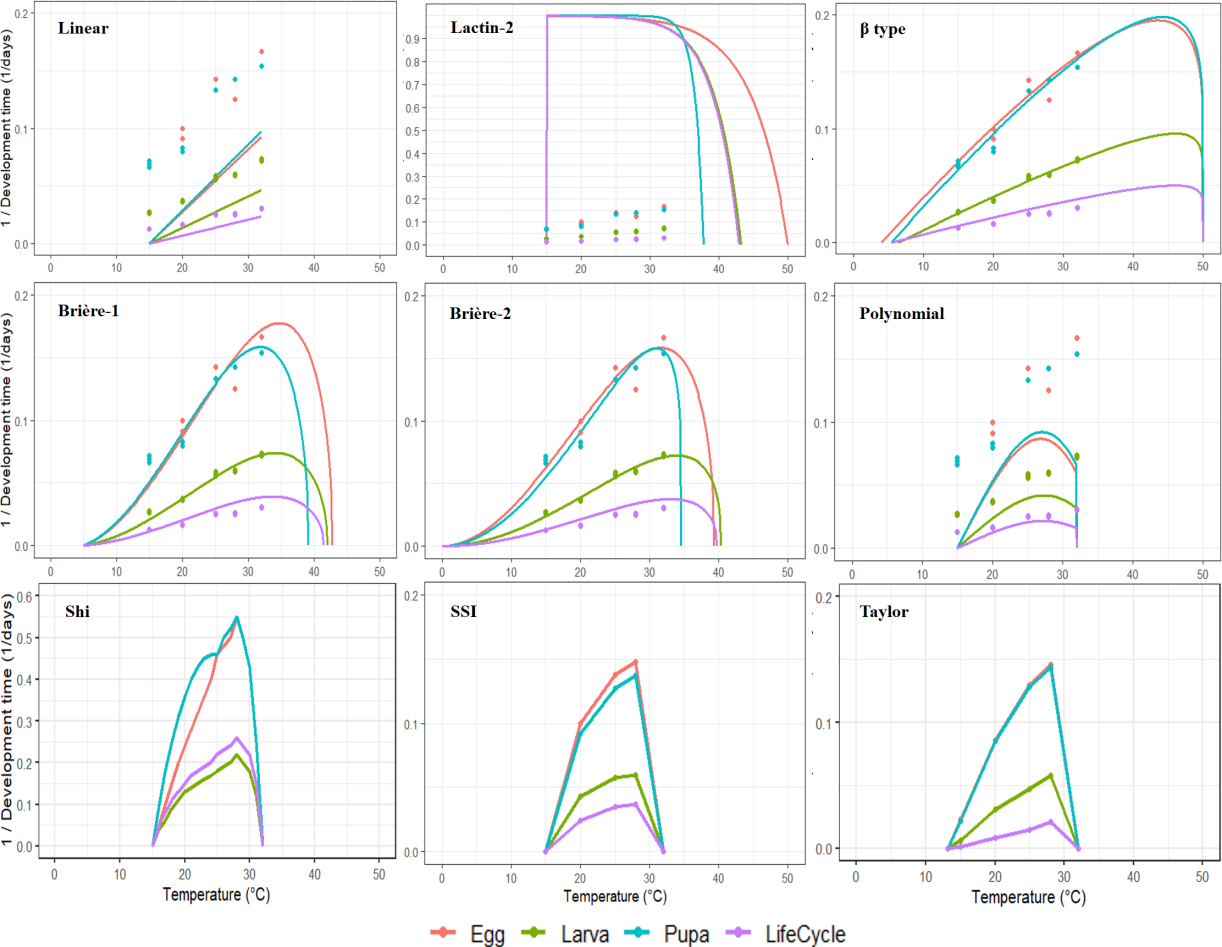
Fitted curves of mathematical models describing the temperature-dependent development rates of *Prays oleae* for egg, larval, pupal stages, and the complete life cycle.

The performance of nine mathematical models describing the temperature-dependent development of *P. oleae* varied across developmental stages (**Tables 5**–**7**, **Figure 3**). Model fit was evaluated using the regression standard error (S) and the corrected Akaike information criterion (ΔAICc). For egg development, Lactin-2 had the lowest ΔAICc (0.0), indicating the best statistical fit, although the standard error (S = 1010 × 10⁻³) was high. Linear (ΔAICc = 57.8, S = 10.8 × 10⁻³), Brière-2 (ΔAICc = 59.1, S = 13.6 × 10⁻³), Brière-1, Taylor, Polynomial, β-type, Shi, and SSI had higher ΔAICc values, reflecting lower relative accuracy. For the SSI model applied to eggs, the number of fitted parameters (k = 7) slightly exceeded the number of temperature treatments minus 1 (n − k − 1 < 0), so AICc should be interpreted cautiously; this model was included for comparison only. Thermal thresholds varied across models: *T_L_* ranged from 1.6 °C (Linear) to 15 °C (SSI), *T_H_* from 37.8 °C (Lactin-2) to 50 °C (β-type), and *T_opt_* from 28–34.8 °C. For larval development, Lactin-2 again performed well (ΔAICc = 2.2, S = 1090 × 10⁻³), followed by Linear (ΔAICc = 15.0, S = 2.6 × 10⁻³) and β-type (ΔAICc = 15.4, S = 2.8 × 10⁻³). Thermal thresholds ranged from *T_L_* = 5.5 °C (Linear) to 16 °C (SSI), *T_H_* from 37.8 °C (Lactin-2) to 43.2 °C (β-type), and *T_opt_* from 29–34 °C. For pupal development, Lactin-2 provided the best fit (ΔAICc = 0.4, S = 1030 × 10⁻³), followed by SSI (ΔAICc = 14.5, S = 135.3 × 10⁻³). Thermal thresholds ranged from *T_L_* = 3.6 °C (Linear) to 15 °C (SSI), *T_H_* from 37.8 °C (Lactin-2) to 50 °C (β-type), and *T_opt_* from 27–31 °C. For the complete life cycle, most models (Linear, β-type, Brière-1, Brière-2, Shi, SSI, Taylor, Polynomial) had ΔAICc = 0, indicating similarly good performance, while Lactin-2 had ΔAICc = 3.0, slightly higher but still acceptable. Thermal thresholds for the full life cycle ranged from *T_L_*  = 4.3 °C (Linear) to 15 °C (SSI), *T_H_* from 39.7 °C (Brière-2) to 42.9 °C (Lactin-2), and *T_opt_* from 28–33 °C. Some models produced unrealistic thresholds, such as Taylor (*T_L_*  = 11.7 °C for larvae) and SSI (*T_H_* exceeding observed temperature range). In contrast, Brière-1, Brière-2, β-type, and Shi consistently produced biologically reasonable thresholds aligned with observed development times and known species distribution (**Table 7**). Considering both goodness-of-fit and accuracy of thermal thresholds, Brière-2 emerged as the most suitable model, followed by Brière-1, β-type, and Shi, for describing the temperature-dependent development of *P. oleae*.

**Table 5:** Comparison of the performance of nine mathematical models describing the temperature-dependent development rate of *Prays oleae* based on regression standard error (S) and corrected Akaike information criterion (AICc).

Model	Egg		Larvae		Pupae		Life span	
	S (10^−3^)	^a^ΔAICc	S(10^−3^)	^a^ΔAICc	S(10^−3^)	^a^ΔAICc	S(10^−3^)	^a^ΔAICc
Linear	10.8	57.8	2.6	15.0	8.9	51.9	1.6	**0.0**
Lactin-2	1010	**0.0**	1090	2.2	1030	**0.4**	1120	**3.0**
β type	11.6	15.8	2.8	15.4	9.5	51.7	1.7	**0.0**
Brière-1	18.5	59.6	4.0	13.6	11.0	44.1	2.5	**0.0**
Brière-2	13.6	59.1	3.1	14.4	10.1	50.2	1.9	**0.0**
Polynomial	28.3	45.2	11.8	19.1	26.7	43.5	6.3	**0.0**
Shi	147.6	44.6	62.2	18.6	142.8	43.6	33.4	**0.0**
SSI	140.5	14.8	59.5	6.2	135.3	14.5	31.9	**0.0**
Taylor	12.6	53.9	3.9	18.6	9.4	45.2	2.1	**0.0**

a ΔAICc values in bold indicate models with similar performance (ΔAICc ≤ 2).

**Table 6:** Parameter (mean) estimates for each mathematical model describing the effect of temperature on the development rate of different life stages of *Prays oleae*.

Model^a^	Egg	Larvae	Pupae	Life span
Linear				
a	-0.0089	-0.0149	-0.0204	-0.0060
b	0.0054	0.0027	0.0057	0.0014
*T_L_*	1.6	5.5	3.6	4.3
*K*	183.6	366.0	174.6	720.1
Lactin-2				
*ρ*	0.120	0.085	0.060	0.040
*Δ*	5.13	3.68	1.31	3.61
λ	-0.050	-0.030	-0.020	-0.025
$T_{L}$	8.0	10.0	12.0	9.0
*T_opt_*	30.0	33.0	30.0	33.0
$T_{H}$	50.0	43.2	37.8	42.9
β type				
ρ	0.0036	0.0021	0.0039	0.0011
α	0.16	0.10	0.15	0.10
*β*	10.6	10.4	11.3	9.53
$T_{L}$	4.1	6.5	5.5	5.5
*T_opt_*	28.0	30.0	27.0	29.0
$T_{H}$	50.0	43.2	37.8	42.9
Brière-1				
a	6.1×10^-5^	2.6×10^-5^	6.8×10^-5^	1.5×10^-5^
$T_{L}$	5.0	5	5	5
*T_opt_*	34.8	34.2	31.9	33.8
$T_{H}$	42.8	42.0	39.2	41.5
Brière-2				
a	5.8×10^-5^	3.1×10^-5^	1.2×10^-4^	1.6×10^-5^
*m*	2.0	2.7	4.3	2.5
$T_{L}$	8.0	9.0	7.5	8.5
*T_opt_*	31.6	34.0	31.0	33.0
$T_{H}$	39.4	40.3	34.6	39.7
Polynomial				
a	-2.8×10^-5^	-1.3×10^-5^	-2.9×10^-5^	-6.7×10^-6^
b	1.3×10^-3^	6.2×10^-4^	1.4×10^-3^	3.2×10^-4^
C	-9.3×10^-3^	-5.1×10^-3^	-1.1×10^-2^	-2.6×10^-3^
d	3.0×10^-3^	1.6×10^-3^	4.2×10^-3^	8.4×10^-4^
*T_opt_*	26.9	27.3	27.1	27.1
Shi				
*k*	-0.05	-0.05	-0.05	-0.05
*m*	9.8×10^-3^	4.4×10^-3^	9.8×10^-3^	2.3×10^-3^
$T_{L}$	12.0	13.0	12.0	12.0
*T_opt_*	25.0	27.0	24.0	26.0
$T_{H}$	32.0	32.0	32.0	32.0
SSI				
*ΔH_A_*	5.0×10^-4^	4.8×10^4^	5.2×10^4^	4.7×10^4^
*ΔH_L_*	-1.0×10^4^	-9.0×10^3^	-9.5×10^3^	-8.5×10^3^
*ΔH_H_*	1.0×10^4^	9.5×10^3^	9.7×10^3^	9.4×10^3^
TL(K)	288.2	289.2	288.2	288.2
TH(K	305.2	307.2	306.2	307.2
$T_{\emptyset }$	32.0	32.0	32.0	32.0
$\rho_{\emptyset }$	0.1667	0.0714	0.1538	0.0377
$T_{L}$	15.0	16.0	15.0	15.0
*T_opt_*	28.0	29.0	27.0	28.0
$T_{H}$	32.0	34.0	33.0	34.0
Taylor				
Rm	0.1551	0.0680	0.1535	0.0361
$T_{L}$	13.2	11.7	12.3	12.2
*T_opt_*	28.5	30.0	29.0	28.8

$T_{L}$, T***_H_*,** and T***_opt_*** represent the lower thermal threshold, upper thermal threshold, and the optimum temperature for development (°C), respectively. *K* denotes the thermal constant. T*_Φ_* is the intrinsic optimum temperature at which the probability of the enzyme being in its active state is maximal (expressed in *K*), and *ρ_Φ_* is the corresponding development rate at *T_Φ_*, assuming no enzyme inactivation. All other variables are parameters estimated through model fitting.

**Table 7:** Thermal thresholds estimated by each model used to describe the temperature-dependent development rate of *Prays oleae*, and their accuracy is inferred based on the observed development times and known species distribution range.

Models	No. of estimated thermal thresholds	Accuracy^a^		
		$T_{L}$	$T_{H}$	*T_opt_*
Linear	1	+	•	•
Lactin-2	3	+	+	−
β type	3	+	+	+
Brière-1	3	+	+	+
Brière-2	3	+	+	+
Polynomial	2	•	−	+
Shi	3	+	+	+
SSI	3	−	−	+
Taylor	2	−	•	+

**ᵃ +**, yes; **−**, no; **•**, parameter not estimated by the model. $T_{L}\;$and *T_H_* are the lower and upper thermal thresholds, respectively, and *T_opt_* is the optimum temperature.

## 4 Discussion

Temperature strongly influenced the development, survival, and reproduction of *Prays oleae*, as observed in other Lepidoptera, including olive pests such as *Palpita unionalis* (Hübner) (Lepidoptera: Pyralidae) (43–45) and generalists like *Spodoptera eridania* (Stoll) (Lepidoptera: Noctuidae) (29, 46, 47). Development accelerated with increasing temperature (15–32 °C), lifespan decreased at higher temperatures, and females developed more slowly than males, particularly in the larval stage. Sex-specific patterns, with females developing more slowly than males, likely result from differential resource allocation, where females invest more time in growth and nutrient storage to enhance fecundity, while males develop faster to increase early mating success. These patterns are consistent with observations in other Lepidoptera, including *P. unionalis* (21, 23, 45, 48). *Prays oleae* was unable to complete development at 35 °C, showing that this temperature exceeds its upper thermal threshold, likely due to protein denaturation, impaired enzyme activity, or hormonal disruption (20, 21). Full development occurred between 15 and 32 °C, with moderate temperatures (20-28 °C) favoring survival and growth. This upper limit is ecologically significant in Mediterranean regions, including Morocco, where high summer temperatures may naturally reduce *P. oleae* populations (49). Knowledge of these thermal limits is essential for predicting pest outbreaks and guiding the timing of monitoring and control measures, especially under climate change scenarios that may shift pest pressure to milder regions. Survival of *P. oleae* followed a bell-shaped pattern, highest at 20–25 °C and sharply reduced at 15 °C and 32 °C, with mean survival time decreasing as temperature increased. Similar bell-shaped survival curves have been documented in *P. unionalis* and *S. eridania* (Lepidoptera: Noctuidae) under laboratory conditions (29, 43, 46). Pupal weight declined at higher temperatures, with females heavier than males, reflecting sex-specific resource allocation. Reduced mass and increased adult deformities at thermal extremes likely result from shortened larval feeding and disrupted metamorphosis (20–22). These patterns highlight the species’ sensitivity to temperature and potential impacts on population growth and reproduction. Female fecundity peaked at 25 °C, declining at lower and higher temperatures, with pre-oviposition shortening at higher temperatures and oviposition longest at 25 °C. These results demonstrate that both extreme cold and heat compromise reproductive output, consistent with studies in *P. unionalis* and other Lepidoptera (50, 51). The alignment of optimum temperature for fecundity (25–28 °C) with highest survival suggests that population growth potential is maximized within this thermal range.

The performance of the mathematical models used to describe the temperature-dependent development rate of *P. oleae* varied widely, highlighting the importance of testing multiple models and applying a multicriteria selection process (31, 52, 53). Our comparison of nine temperature-dependent development models revealed that nonlinear models, particularly Brière-2, Brière-1, β-type, and Shi, best described development across stages and produced biologically reasonable thermal thresholds. Most models had (n − k − 1) positive, ensuring valid AICc calculations, with the exception of the SSI model applied to eggs (k = 7, n − k − 1 < 0), for which the AICc should be interpreted cautiously. Several models showed ΔAICc = 0, not because their performance was identical, but due to the small number of temperature treatments per stage (n = 5–6), which limits the effective sample size and produces very small differences in AICc that were rounded in **Table 5**. This underscores the importance of evaluating both statistical fit and biological plausibility when selecting models, rather than relying solely on ΔAICc. The linear model underestimated upper thresholds, while SSI and Taylor models occasionally predicted unrealistic temperatures. These results align with the literature, which emphasizes the superior performance of nonlinear models in capturing the asymmetric, curvilinear nature of temperature-development relationships in insects (24, 26–29). This approach reinforces the notion that selecting models solely based on statistical performance can be misleading; including accuracy of thermal thresholds provides fundamental information for predicting insect occurrence under field conditions (46, 54). Since their introduction in 1995 and 1999, the Brière-2 and Lactin-2 models have been widely applied to describe the development rates of arthropods (29). These models have demonstrated consistent suitability across a variety of species (53, 55–58). In contrast, the model developed by Shi et al. (40) has been applied in relatively few studies, likely due to its more recent publication. Thermal thresholds of *P. oleae* varied among models and stages: pupal *T_L_* ranged from 3.6 °C (Linear) to 15 °C (SSI), egg–adult *T_L_* from 4.3 °C to 15 °C, *T_opt_* from 24 °C (Shi, pupae) to 34 °C (Brière-2, larvae), and *T_H_* from 32 °C (Shi) to 50 °C (β-type, eggs). Development failed at 35 °C, indicating this exceeds the species’ upper limit. Moderate temperatures of 20–30 °C support optimal development, while below 10–15 °C or above 34–35 °C restrict growth. Overall, the optimal temperature is 27–29 °C. This is consistent with previous observations in related Lepidoptera (43, 46). The *T_Φ_* from the SSI model indicates the temperature of maximal enzyme activity, reflecting optimal conditions for development, survival, and reproduction. Estimated *T_Φ_* values of 32 °C for larval, pupal, and egg–adult stages support an overall optimal range of 25–32 °C for *P. oleae*, consistent with other Lepidoptera such as *P. unionalis* and *S. eridania*, which also perform best at moderate temperatures (25-30 °C) and show sharply reduced survival at thermal extremes (15–40 °C) (29, 48). Comparisons among studies should be conducted carefully due to methodological differences, including the rearing substrate and the temperature range assessed. In our study, we reared *P. oleae* on olive leaves under controlled laboratory conditions, whereas other studies on related Lepidoptera often used different host plants or artificial diets (43, 46). Furthermore, we evaluated *P. oleae* development over a broader temperature range (15–35 °C) than most previous studies, which allowed us to capture both the lower and upper thermal thresholds for development. These methodological differences likely contributed to variation in estimated thermal parameters. For instance, the lower developmental threshold (*T_L_*) estimated in this study for the egg stage was 1.6 °C (Linear model), which is lower than the *T_L_* reported for other Lepidoptera such as *S. eridania* (10.8 °C) (29, 48). Similarly, the thermal constant (K) for the egg–adult cycle of *P. oleae* ranged from 174.6 to 720.1 degree-days depending on the model, reflecting the influence of methodological and modeling choices on parameter estimation. While it remains unclear whether these differences are primarily due to the range of temperatures tested or the host plant used, it is widely recognized that evaluating insect development across a broad temperature range yields more robust and reliable estimates of thermal thresholds (59). The selected mathematical models can be applied to pest management programs by simulating *P. oleae* development under field conditions and predicting periods of high vulnerability for control interventions. Accurate modeling may help optimize the timing of biological or chemical control applications, reducing environmental impact and increasing efficacy, as successfully demonstrated in other Lepidoptera such as *Tuta absoluta* (Meyrick) (Lepidoptera: Gelechiidae), and *Grapholita molesta* (Busck) (Lepidoptera: Tortricidae) (28, 33). However, field validation is necessary, particularly because model predictions depend on accurate host plant phenology, initial population observations, and local temperature fluctuations (29). Thermal thresholds and *TΦ* values suggest optimal performance at 25–32 °C, with sharply reduced survival and reproduction at extremes, offering mechanistic insights into temperature-dependent population dynamics. By integrating broad-range thermal responses with multiple model comparisons, this study provides novel predictive tools for simulating *P. oleae* phenology and optimizing the timing of management interventions. Future field validation should examine cultivar- and microclimate-specific effects to refine model predictions under natural conditions.

## 5 Conclusion

Our study demonstrates that the development and survival of *P. oleae* are strongly influenced by temperature. Model-based estimates of thermal thresholds indicate that the species has a lower developmental limit (*T_L_*) ranging from 1.6–15.0 °C depending on life stage and model, an optimum temperature (*T_opt_*) between 27.0 and 34.0 °C, and an upper thermal threshold (*T_H_*) of 32.0–50.0 °C. P. oleae failed to complete development at 35 °C, confirming that temperatures above this limit are detrimental to its survival and development. Moderate temperatures, approximately 20–30 °C, provide the most favorable conditions for growth, survival, and reproduction. Among the models tested, nonlinear models such as Brière-2, Lactin-2, and Shi provided the most reliable estimations of thermal thresholds across life stages. These findings have practical implications for predicting population dynamics and improving the timing of integrated pest management strategies in Mediterranean olive-growing regions, including Morocco, where temperature extremes may naturally limit *P. oleae* populations.

## Data Availability

The original contributions presented in the study are included in the article/supplementary material. Further inquiries can be directed to the corresponding author.

## References

[1] Bernard PJ . Section II. Des insectes qui vivent sur l’olivier. In: Mémoires pour servir à l’histoire naturelle de la Provence, vol. 2. Didot fils aîné, Paris (1788). p. 265–319.

[2] Alves JF Mendes S Alves da Silva A Sousa JP Paredes D . Land-use effect on olive groves pest *Prays oleae* and on its potential biocontrol agent. Chrysoperla carnea. Insects. (2021) 12:46. doi: 10.3390/insects12010046, PMID: 33435550 PMC7827753

[3] Kos T Zdrilić A Čirjak D Zorica M Kolega Š Pajač Živković I . Towards smart pest management in olives: ANN-based detection of olive moth (Prays oleae Bernard 1788). AgriEngineering. (2025) 7:200. doi: 10.3390/agriengineering7070200, PMID: 41725453

[4] Lantero E Matallanas B Callejas C . Current status of the main olive pests: Useful integrated pest management strategies and genetic tools. Appl Sci. (2023) 13:12078. doi: 10.3390/app132112078, PMID: 41725453

[5] Tiring G Ada M Ada M Dona R Kalkan Ç. Satar S . The population fluctuation of *Prays oleae* Bern (Lepidoptera: Praydidae, Yponomeutidae) in three different olive orchards. Çukurova Tarım ve Gıda Bilimleri Dergisi. (2024) 39:367–74. doi: 10.36846/CJAFS.2024.156

[6] Armendàriz I De La Iglesia Santiago Y Campillo G Alberte C Miranda L . Ciclo del Prays del olivo (Prays oleae Bern.) en Arribes del Duero. Boletin Sanidad Vegetal. Plagas. (2007) 33:443–55.

[7] Villa M Santos SA Sousa JP Ferreira A da Silva PM Patanita I . Landscape composition and configuration affect the abundance of the olive moth (Prays oleae, Bernard) in olive groves. Agriculture Ecosyst Environ. (2020) 294:106854. doi: 10.1016/j.agee.2020.106854, PMID: 41802445

[8] Ramos P Campos M Ramos JM . Long-term study on the evaluation of yield and economic losses caused by *Prays oleae* Bern. in the olive crop of Granada (southern Spain). Crop Prot. (1998) 17:645–7. doi: 10.1016/S0261-2194(98)00065-9, PMID: 41276264

[9] Çetin H Alaoğlu Ö . Mut (Mersin) İlçesinde Zeytin güvesi (*Prays oleae* Bern.) (Lepidoptera: Yponomeutidae)’nin popülasyon değişimi ve zararı üzerinde araştırmalar. Turkish J Entomology. (2005) 29:125–34.

[10] Broumas T Haniotakis G Liaropoulous C Tomazou T Ragoussis N . The efficacy of an improved form of the mass-trapping method for the control of the olive fruit fly, *Bactrocera oleae* (Gmelin) (Dipt., Tephritidae): pilot-scale feasibility studies. J Appl Entomology. (2002) 126:217–23. doi: 10.1046/j.1439-0418.2002.00637.x, PMID: 41717205

[11] Delrio G . Biological control of olive pests in the Mediterranean region. Integrated Protection of Olive Crops. WPRS Bull. (2010) 53:85–92.

[12] Hilal A Ouguas Y . Integrated control of olive pests in Morocco. IOBC WPRS Bull. (2005) 28:101.

[13] Álvarez HA Jiménez-Muñoz R Morente M Campos M Ruano F . Ground cover presence in organic olive orchards affects the interaction of natural enemies against Prays oleae, promoting an effective egg predation. Agriculture Ecosyst Environ. (2021) 315:107441. doi: 10.1016/j.agee.2021.107441, PMID: 41802445

[14] Collier TR Van Steenwyk RA . Prospects for integrated control of olive fruit fly are promising in California. California Agric. (2003) 57:28–31. doi: 10.3733/ca.v057n01p28, PMID: 41029052

[15] Haniotakis GE . Olive pest control: present status and prospects. IOBC WPRS Bull. (2005) 28:1.

[16] Porcel M Campos M Ruano F Sanllorente O Caballero JA . Incidence of the OLIPE mass-trapping on olive non-target arthropods. Spanish J Agric Res. (2009) 7:660–4. doi: 10.5424/sjar/2009073-459

[17] Ait Mansour A Ouanaimi F Chemseddine M Boumezzough A . Study of the flight dynamics of *Prays oleae* (Lepidoptera: Yponomeutidae) using sexual trapping in olive orchards of Essaouira region, Morocco. J Entomology Zoology Stud. (2017) 5:943–52.

[18] Alexandrakis V Neuenschwander P . Le rôle d'*Aphytis Chilensis* [Hym.: Aphelinidae], parasite d'*Aspidiotus nerii* [Hom.: Diaspididae] sur olivier en Crète. Entomophaga. (1980) 25:61–71. doi: 10.1007/BF02377523, PMID: 41804457

[19] Civantos-Ruiz M Gómez-Guzmán JA Sainz-Pérez M González-Ruiz R . Application of accumulated heat units in the control of the olive moth, *Prays oleae* (Bern.) (Lep. Praydidae), in olive groves in southern Spain. Rev Bras Fruticultura. (2022) 44:1–20. doi: 10.1590/0100-29452022804, PMID: 41801159

[20] Denlinger DL Yocum GD . Physiology of heat sensitivity. Boulder, CO: Westview Press (1998) p. 7–57.

[21] Porter JH Parry ML Carter TR . The potential effects of climatic change on agricultural insect pests. Agric For Meteorology. (1991) 57:221–40. doi: 10.1016/0168-1923(91)90088-8

[22] Bale JS Masters GJ Hodkinson ID Awmack C Bezemer TM Brown VK . Herbivory in global climate change research: Direct effects of rising temperature on insect herbivores. Global Change Biol. (2002) 8:1–16. doi: 10.1046/j.1365-2486.2002.00451.x, PMID: 41717205

[23] Netherer S Schopf A . Potential effects of climate change on insect herbivores in European forests—general aspects and the pine processionary moth as specific example. For Ecol Manage. (2010) 259:831–8. doi: 10.1016/j.foreco.2009.07.034, PMID: 41802445

[24] Damos P Savopoulou-Soultani M . Temperature-driven models for insect development and vital thermal requirements. Psyche: A J Entomology. (2012) 1:123405.

[25] Ramos P Campos M Ramos JM . Influencia de los factores ambientales sobre la mortalidad de larvas y crisálidas de *Prays oleae* Bern. (Lepidoptera: Plutellidae). Boletin la Asociación Española Entomología. (1978) 2:143–7.

[26] Logan JA Wollkind DJ Hoyt SC Tanigoshi LK . An analytic model for description of temperature-dependent rate phenomena in arthropods. Environ Entomology. (1976) 5:1133–40. doi: 10.1093/ee/5.6.1133, PMID: 34916285

[27] Lactin DJ Holliday NJ Johnson DL Craigen R . Improved rate model of temperature-dependent development by arthropods. Environ Entomology. (1995) 24:68–75. doi: 10.1093/ee/24.1.68

[28] Marchioro CA Krechemer FS Foerster LA . Estimating the development rate of the tomato leaf miner, *Tuta absoluta* (Lepidoptera: Gelechiidae), using linear and non-linear models. Pest Manage Sci. (2017) 73:1486–93. doi: 10.1002/ps.4484, PMID: 27860137

[29] Sampaio F Krechemer FS Marchioro CA . Temperature-dependent development models describing the effects of temperature on the development of *Spodoptera eridania*. Pest Manage Sci. (2021) 77:919–29. doi: 10.1002/ps.6101, PMID: 32975885

[30] Ikemoto T Takai K . A new linearized formula for the law of total effective temperature and the evaluation of line-fitting methods with both variables subject to error. Environ Entomology. (2000) 29:671–82. doi: 10.1603/0046-225X-29.4.671, PMID: 38536611

[31] Quinn BK . A critical review of the use and performance of different function types for modeling temperature-dependent development of arthropod larvae. J Thermal Biol. (2017) 63:65–77. doi: 10.1016/j.jtherbio.2016.11.013, PMID: 28010817

[32] Marchioro CA Krechemer FS Moraes CP Foerster LA . A stochastic model for predicting the stage emergence of *Plutella xylostella* under field conditions. Ann Appl Biol. (2016) 169:190–9. doi: 10.1111/aab.12290, PMID: 41803443

[33] Ahn JJ Yang CY Jung C . Model of *Grapholita molesta* spring emergence in pear orchards based on statistical information criteria. J Asia-Pacific Entomology. (2012) 15:589–93. doi: 10.1016/j.aspen.2012.04.002, PMID: 41802445

[34] Hanula JL DeBarr GL Weatherby JC Barber LR Berisford CW . Degree-day model for timing insecticide applications to control *Dioryctria amatella* (Lepidoptera: Pyralidae) in loblolly pine seed orchards. Can Entomologist. (2002) 134:255–68. doi: 10.4039/Ent134255-2

[35] Shehata WA Abou-Elkhair SS Youssef AA Nasr FN . Biological studies on the olive leaf moth, *Palpita unionalis* Hübner (Lepidoptera: Pyralidae), and the olive moth, *Prays oleae* Bernard (Lepidoptera: Yponomeutidae). J Pest Sci. (2003) 76:155–8. doi: 10.1007/s10340-003-0011-8, PMID: 41804457

[36] Rodrigues A Batista V Nave A Matos C da Costa ECA . Contribution for the development of a degree-day model for the olive moth, *Prays oleae* (Bernard). Rev Ciências Agrárias. (2017) 40:111–8. doi: 10.19084/RCA16185

[37] Campbell A Frazer BD Gilbert N Gutierrez AP Mackauer M . Temperature requirements of some aphids and their parasites. Journal of Applied Ecology. (1974) 11:419–423. doi: 10.2307/2402197, PMID: 39964225

[38] Brière JF Pracros P Le Roux AY Pierre JS . A novel rate model of temperature-dependent development for arthropods. Environmental Entomology. (1999) 28:22–29. doi: 10.1093/ee/28.1.22

[39] Lamb RJ Gerbey GH Atkinson GF . Comparison of developmental rate curves applied to egg hatching data of Entomoscelis americana Brown (Coleoptera: Chrysomelidae). Environmental Entomology. (1984) 13:868–872. doi: 10.1093/ee/13.3.868

[40] Shi P Ge F Sun Y Chen C . A simple model for describing the effect of temperature on insect developmental rate. J Asia-Pacific Entomology. (2011) 14:15–20. doi: 10.1016/j.aspen.2010.11.008, PMID: 41802445

[41] Ikemoto T Kurahashi I Shi PJ . Confidence interval of intrinsic optimum temperature estimated using thermodynamic SSI model. Insect Sci. (2013) 20:420–8. doi: 10.1111/j.1744-7917.2012.01525.x, PMID: 23955893

[42] Taylor F . Ecology and evolution of physiological time in insects. The American Naturalist. (1981) 117:1–23.

[43] Alexopoulou-Vassilaina P Santorini AP . Some data on the biology of *Palpita unionalis* Hb. (Lepidoptera: Pyralidae) under laboratory conditions. Annales l’Institut Phytopathologique Benaki N.S. (1973) 10:320–6.

[44] Amin AH Saleh MRA . Seasonal activity of the olive leaf moth, *Palpita unionalis* (Hübner) (Lepidoptera: Pyralidae), in Kharga Oasis, New Valley, Egypt. Ann Agric Science Faculty Agriculture Ain Shams Univ. (1975) 20:35–41.

[45] Badaawi A Awadallah AM Foda SM . On the biology of the olive leaf moth *Palpita unionalis* Hübner (Lepidoptera: Pyralidae). Z für Angewandte Entomologie. (1976) 81:103–10.

[46] Montezano DG Specht A Sosa–Gomez DR Roque–Specht VF de Barros NM . Immature stages of *Spodoptera eridania* (Lepidoptera: Noctuidae): developmental parameters and host plants. J Insect Sci. (2014) 14:238. doi: 10.1093/jisesa/ieu100, PMID: 25525103 PMC5634020

[47] Capinera JL . Southern armyworm, *Spodoptera eridania* (Stoll) (Insecta: Lepidoptera: Noctuidae) (2020). Available online at: http://entnemdept.ufl.edu/creatures/veg/leaf/southern_armyworm.htm (Accessed 25 2020).

[48] El-Kifl AH Abdel-Salam AL Rahhal AMM . Biological studies on the olive leaf moth, *Palpita unionalis* Hübner (Lepidoptera: Pyralidae). Bull la Société Entomologique d’Égypte. (1974) 58:337–44.

[49] El Aalaoui M Mokrini F Sbaghi M . Impact of temperature on development and reproduction of the olive black scale *Saissetia oleae* (Olivier) (Hemiptera: Coccidae). Bull Entomological Res. (2025) 115:296–307. doi: 10.1017/S0007485325000112, PMID: 40025983

[50] El-Hakim AM Kisk SA . Cultural methods for the control of olive pests. Bulletin Faculty Agriculture Cairo Univ. (1988) 39:345–51.

[51] El-Khawas MA . Integrated control of insect pests on olive trees in Egypt with emphasis on biological control. Faculty of Science, Cairo University, Egypt (2000). 247 pp.

[52] Angilletta MJ . Estimating and comparing thermal performance curves. J Thermal Biol. (2006) 31:541–5. doi: 10.1016/j.jtherbio.2006.06.002, PMID: 41802445

[53] Jacques J Sampaio F Santos HT Marchioro CA . Climate change and voltinism of Mythimna sequax: the location and choice of phenological models matter. Agric For Entomology. (2019) 21:431–44. doi: 10.1111/afe.12350, PMID: 41803443

[54] Foerster LA Dionísio ALM . Temperature requirements for the development of *Spodoptera eridania* (Cramer 1782) (Lepidoptera: noctuidae) on “bracatinga” (Mimosa scabrella bentham) (Leguminosae). Annales la Société Entomologique du Brésil. (1989) 18:145–54.

[55] Roy M Brodeur J Cloutier C . Relationship between temperature and developmental rate of *Stethorus punctillum* (Coleoptera: Coccinellidae) and its prey *Tetranychus mcdanieli* (Acarina: Tetranychidae). Environ Entomology. (2002) 31:177–87. doi: 10.1603/0046-225X-31.1.177, PMID: 38536611

[56] Kontodimas DC Eliopoulos PA Stathas GJ Economou LO . Comparative temperature-dependent development of *Nephus includens* (Kirsch) and *Nephus bisignatus* (Boheman) (Coleoptera: Coccinellidae) preying on *Planococcus citri* (Risso) (Homoptera: Pseudococcidae): Evaluation of a linear and various nonlinear models using specific criteria. Environ Entomology. (2004) 33:1–11. doi: 10.1603/0046-225X-33.1.1, PMID: 38536611

[57] Haghani M Fathipour Y Talebi AA Baniameri V . Temperature dependent development of *Diglyphus isaea* (Hymenoptera: Eulophidae) on *Liriomyza sativae* (Diptera: Agromyzidae) on cucumber. J Pest Sci. (2007) 80:71–7. doi: 10.1007/s10340-006-0154-5, PMID: 41804457

[58] Sánchez-Ramos I Pascual S Fernández CE Marcotegui A González-Núñez M . Effect of temperature on the survival and development of the immature stages of *Monosteira unicostata* (Hemiptera: Tingidae). Eur J Entomology. (2015) 112:664–75. doi: 10.14411/eje.2015.087

[59] Dixon AF Honek A Keil P Kotela MAA Šizling AL Jarošík V . Relationship between the minimum and maximum temperature thresholds for development in insects. Functional Ecology. (2009) 23:257–264.

